# Harnessing the power of thermosensitive liposomes with gold nanoprisms and silica for controlled drug delivery in combined chemotherapy and phototherapy†

**DOI:** 10.1039/d4ra03359k

**Published:** 2024-07-22

**Authors:** Marta Rubio-Camacho, Carlos Cuestas-Ayllón, Beatriz Torres-Herrero, María José Martínez-Tomé, Jesús M. de la Fuente, C. Reyes Mateo

**Affiliations:** a Instituto de Investigación, Desarrollo e Innovación en Biotecnología Sanitaria de Elche (IDiBE), Universidad Miguel Hernández (UMH) c/Avenida de la Universidad de Elche s/n 03202 Elche Alicante Spain marta.rubioc@umh.es; b Instituto de Nanociencia y Materiales de Aragón (INMA), CSIC-Universidad de Zaragoza (UNIZAR), CIBER-BBN c/Pedro Cerbuna s/n 50009 Zaragoza Spain

## Abstract

In recent years, the scientific community has tried to address the treatment of complex diseases such as cancer in a more appropriate and promising way. Regarding this and benefiting from the unique optical properties of gold nanoprisms (AuNPRs), the physicochemical properties of thermosensitive liposomes (TSLs), and the tunable drug encapsulation and release properties of silica nanoparticles (BioSi@NPs), this study has developed two nanoformulations. These nanoformulations have the potential to integrate chemotherapy and photothermal therapy within a single entity. Once their components were synthesized and characterized separately, two strategies were taken in order to develop these multifunctional nanoformulations: (1) covalent binding of AuNPRs to TSLs and (2) co-encapsulation of both components within BioSi@NPs, without modifying the optical and physicochemical properties of AuNPRs and TSLs. Finally, the suitability of both nanoformulations to carry and release hydrophilic drugs when triggered by a 1064 nm NIR laser has been explored by using the fluorescent probe 5(6)-carboxyfluorescein (CF) as a hydrophilic drug model. Different laser power and time of exposure were also tested evidencing that hydrophilic drugs were only released from TSLs in the presence of AuNPRs and that the drug release profile was dependent on the type of nanoformulation and irradiation conditions used. In conclusion, these multifunctional nanoformulations exhibit promising potential for controlled drug delivery in combined chemotherapy and phototherapy, with the capability to precisely control the release kinetics based on specific therapeutic needs.

## Abstract 2

Strategies used to obtain multifunctional nanoformulations for controlled drug delivery in combined chemotherapy and phototherapy.

## Graphical abstract

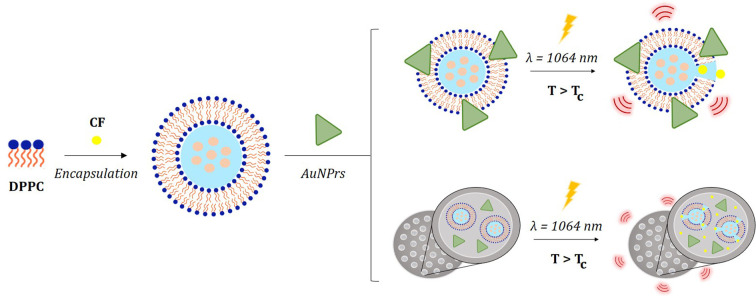

## 1 Introduction

In recent years, nanotechnology has experienced rapid development owing to its wide range of applications in different fields of knowledge such as medicine, biosensors, food industry, environmental remediation, renewable energy, among others.^1–5^ This huge growth is due to the unique physicochemical properties of nanomaterials, which are quite different from those of the macrometric scale, enabling the synthesis of entirely new materials, devices, systems, and applications.

Among all these materials, gold nanoparticles (AuNPs) stand out for their excellent biocompatibility, inert nature, minimal toxicity, strong light scattering and/or absorbance, ease of synthesis, and functionalizable surface, which have made them potential candidates for their use in biomedicine applications, including bioimaging, sensing, and therapeutics.^6–10^ Among all their properties, it is worth mentioning their unique optical properties caused by their strong interaction with light. The conduction electrons on the metal surface on the nanoparticles undergo a collective oscillation when they are excited by light at specific wavelengths. This oscillation is known as local surface plasmon resonance (LSPR), and it causes the absorption and scattering intensities of AuNPs to be much higher than those of identically sized non-plasmonic nanoparticles.^11,12^ This absorbed light is subsequently transformed into heat through several non-radioactive processes, enabling local hyperthermia and the ablation of several tumors.^13,14^ This property, as well as the photostability and biocompatibility of these nanoparticles, has made them excellent candidates for photothermal therapy (PTT).^12–14^

The effectiveness of PTT is highly dependent on the penetration grade. The lack of efficacy due to this dependency has been improved by the employment of near-infrared (NIR) wavelength lasers which, in addition to enhancing PTT penetrations, avoid both damaging the surrounding tissues by the exposure to energetic radiations as well as being absorbed by certain compounds of the organism.^15,16^ In this sense, the plasmonic band of AuNPs can be easily adjusted to NIR region by controlling physicochemical parameters of gold nanoparticles such as size and shape during their synthesis process.^12^ As a general rule, the plasmonic band of gold nanospheres is located in the visible region of the electromagnetic spectrum, slightly varying its position by a couple of nanometers as a function of their size.^12^ Nonetheless, when gold nanospheres grow asymmetrically, forming anisotropic structures such as gold nanoprisms, gold nanorods, or gold nanoclusters, the plasmonic band is red-shifted from visible to NIR region with increasing sizes.^12,17^ It should be noted that in comparison to nanorods and other anisotropic nanostructures, nanoprisms (AuNPRs) stand out for their ability to be synthesized with a thin structure, ensuring active participation of all metal atoms on their surface in plasmon resonance. This unique property leads to the highest LSPR peak intensities per metal atom compared to other morphologies. Consequently, AuNPRs allow working at very low concentrations with notable photothermal effects.^18^

Chemotherapy and PTT are some of the most widely used combination therapies that have shown promising results in cancer treatments.^19–21^ Although AuNPs exhibit an easily tunable surface for drug binding, higher drug-loading structures are required to achieve an optimal therapeutic effect. In this sense, liposomes are considered one of the most successful nanocarriers for drug delivery in chemotherapy due to their ability to carry both, hydrophilic and hydrophobic drugs in the aqueous core and the lipid bilayer, respectively.^22^ Furthermore, these systems are able to protect the encapsulated drugs since lipid membranes behave as physical barriers, avoiding the release of drugs contained within them as well as the entrance of foreign compounds.^22^ The release of drugs from liposomes can be induced through different stimuli capable of modifying the physical properties of lipid membranes such as temperature, light, pH, ions, magnetic fields, or pressure, among others.^23^ Liposomes able to release their content at a specific temperature are known as thermosensitive liposomes (TSLs) and these structures stand out for the difference in their permeability during the gel phase, in which lipids are tightly packed and lipid membrane exhibit low permeability to molecules, and the fluid phase, in which lipids are more flexible allowing the release biomedicine applications. The lipids most used have their *T*_m_ around the mild hyperthermia temperature (39–43 °C) such as 1,2-dipalmitoyl-*sn*-glycero-3-phospho-rac-(1-glycerol) sodium salt (DPPG) or 1,2-dipalmitoyl-*sn*-glycero-3-phosphocholine (DPPC) (*T*_m_ = 41 °C) alone or in presence of 1,2-distearoyl-*sn*-glycero-3-phosphocholine (DSPC) (*T*_m_ = 55 °C), which increases the final *T*_m_ of the TSLs to 42.5–44.5 °C.^24^

The release of drugs is a crucial aspect in the design of pharmaceutical formulations as it determines the effectiveness of the treatment, the onset and duration of the effect, the minimization of side effects, the improvement of bioavailability, and more.^25^ There are different types of controlled drug release, including immediate, sustained or prolonged, programmed or pulsatile, among others. The choice of which type to use depends on the desired effect. In some cases, a medication must act quickly to relieve symptoms or treat an acute medical condition. Immediate release allows the active ingredients to be rapidly absorbed into the bloodstream, providing a faster effect. However, for certain chronic conditions or diseases requiring long-term treatment, it is important to maintain constant levels of the drug in the body. Sustained release helps achieve this by avoiding peaks and valleys in drug concentration in the blood. In any case, controlled release helps improve treatment effectiveness, as this method aims to decrease the amount and the frequency of administration, minimizing adverse side effects and lowering drug toxicity.^25^

**Scheme 1:**
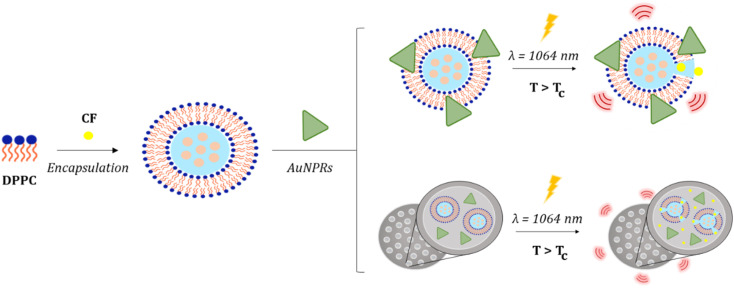
Schematic representation of strategies used in this work to obtain multifunctional nanoformulations.

In the present work, we have benefited from the unique physicochemical properties of TSLs and AuNPRs to develop multifunctional nanoformulations that could potentially integrate chemotherapy and PTT. The high efficiency of light-to-heat conversion of AuNPRs, apart from inducing local hyperthermia, was used to promote the phase transition of TSLs to a high permeability state in which the transported drugs can be released in a controlled manner.^26–29^ In order to integrate both components into a single entity, two strategies were considered: covalently bond the TSLs and AuNPRs, or coencapsulate them avoiding the binding between the two components (Scheme 1). For coencapsulation, silica nanoparticles were used due to their interesting and unique properties such as biocompatibility, tunable size and shape, high surface area, stability, ease of functionalization, and low toxicity, but above all, because of their porosity which provides the system with a high entrapment efficiency and more sustained release of drugs over time.^30,31^ Conventional approaches to synthesizing these types of nanoparticles frequently involve time-consuming procedures, the use of organic surfactants and/or solvents, elevated temperatures, and other hard processing conditions.^32^ Therefore, in this work we have used biomimetic approaches for the synthesis of silica nanoparticles due to the relatively gentle physical and environmentally friendly conditions.^33–35^ These mild reaction conditions associated with biomimetic silica formation allow for the immobilization and entrapment of any molecule present in the synthetic reaction mixture. Indeed, the encapsulation of biomolecules within biomimetic silica particles has been explored for a broad range of enzymes and has demonstrated success in co-entrapping different enzymatic functionalities.^36,37^

Finally, the suitability of these multifunctional systems to release drugs in response to NIR laser irradiation has been analyzed by using the hydrophilic drug model carboxyfluorescein (CF). Furthermore, the release kinetics of these multifunctional nanoformulations were also explored and attempted to modify in order to achieve immediate or sustained release over time, by varying the laser power and time of irradiation. The results of these drug release characterization experiments allowed the selection of the most adequate nanoformulation for the desired purpose.

## 2 Experimental section

### 2.1. Materials

The phospholipid employed in the preparation of TSLs, 1,2-dipalmitoyl-*sn*-glycero-3-phosphocholine (DPPC), as well as the compound used for providing TSLs with positive charge, oleylamine, were purchased from Merck Life Science (Madrid, ES) and used in their original form.

The chemical reagents used for the synthesis of AuNPRs, hydrogen tetrachloroaurate (III) hydrate (HAuCl_4_·H_2_O) and sodium thiosulfate (Na_2_S_2_O_3_), were purchased from Merck Life Science (Madrid, ES), while potassium iodine (KI) was supplied by Cymit Química (Barcelona, ES).

The chemical reagents employed for the pegylation of AuNPRs, sodium borohydride (NaBH_4_) and sodium hydroxide (NaOH), were acquired from Merck Life Science (Madrid, ES), while HS-C_2_H_4_-CONH-PEG-O-C_3_H_6_-COOH (HS-PEG-COOH) (*M*_w_ [g mol^−1^] = 5000) was purchased from Rapp Polymere (Tuebingen, GE).

The chemical reagents used for the conjugation of AuNPRs with TSLs, n-hydroxysulfosuccinimide sodium salt (sulfo-NHS) and 1-ethyl-3-[3-dimethylaminopropyl] carbodiimide hydrochloride (EDC), were supplied by Merck Life Science (Madrid, ES), while 2-(*n*-morpholino)ethanesulfonic acid (MES) was acquired from Alpha Aesar (Kandel, GE).

The chemical reagents used for the synthesis of biomimetic silica particles (BioSi@NPs), polyethylenimine (PEI) (*M*_w_ [g mol^−1^] = 1300), tetramethylorthosilicate (TMOS) and hydrochloric acid (HCl), were all provided by Merck Life Science (Madrid, ES).

The hydrophilic drug model 5(6)-carboxyfluorescein (CF) and the fluorescent membrane probe 1,6-diphenyl-1,3,5-hexatriene (DPH) were obtained from Merck Life Science (Madrid, ES). Stock solutions of these compounds were formulated in dimethyl sulfoxide (DMSO) (1.25 M) and dimethylformamide (DMF) (1 mM), respectively.

Phosphate buffer (50 mM, with and without 0.1 M NaCl, pH 7.4–8) and MES buffer (10 mM, pH 6) were prepared using water deionized and distilled twice with milliQ equipment (Madrid, ES). All other chemicals were of either spectroscopic or analytical reagent grade.

### 2.2. Synthesis of TSLs and CF-loaded TSLs

TSLs were prepared by thin-film hydration method, following the procedure outlined in Rubio-Camacho *et al.*^24^ In brief, lipid solutions of DPPC were allowed to air-dry until CHCl_3_ had completely evaporated. In the case of oleylamine-modified TSLs, this compound was introduced at the beginning of the process in a molar ratio of lipid to oleylamine of 4 : 1. Once the phospholipids were dried, they were reconstituted by vortexing them with Milli-Q water or phosphate buffer, always maintaining the temperature above their transition temperature (∼41 °C), resulting in the formation of multilamellar vesicles (MLVs). Finally, the phospholipid solutions underwent a pressure extrusion process through 0.1 μM polycarbonate filters in order to obtain large unilamellar vesicles (LUVs), again performed above the transition temperature.

The encapsulation of CF on TSLs was carried out as is outlined in Rubio-Camacho *et al.*^24^ In brief, a dried solution of DPPC was reconstituted by vortexing it with a solution of CF 40 mM in phosphate buffer instead of milliQ water or phosphate buffer. This process was carried out maintaining the temperature consistently above the transition temperature of DPPC (∼41 °C). Any non-encapsulated CF was subsequently eliminated using a gel filtration column of Shepadex G-75, eluted with phosphate buffer. Ultimately, the phospholipid solution underwent a pressure extrusion process through 0.1 μM polycarbonate filters, once again above ∼41 °C. The quantification of CF release percentage was carried out by inducing the breakdown of lipid vesicles with Tween20.

### 2.3. Synthesis of AuNPRs and PEGylated AuNPRs

A complex methodology was employed in order to synthesize gold nanoprisms as is described in Ramírez-Jiménez *et al.*^38^ This protocol was previously adapted from Alfranca *et al.*^39^ In brief, HAuCl_4_ (2 mM), Na_2_S_2_O_3_ (0.5 mM), and KI (0.1 M) solutions were prepared with milliQ water. From these solutions, we prepared M1 (150 mL of Na_2_S_2_O_3_) and M2 (240 mL Na_2_S_2_O_3_ + 20 μL of KI). Once all the solutions were prepared, 140 mL of M2 were slowly poured into 200 mL of HAuCl_4_. After 4 minutes of incubation, another 140 mL of M2 were added to the previous solution and it was incubated for 4 minutes. Finally, 60 mL of M1 were also added and the final mixture was left reacting for 1 h. Stepwise additions, as well as incubating periods, were done at 18 °C and protected from light.

**Scheme 2:**
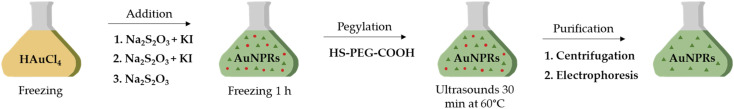
Schematic representation of the synthesis, pegylation, and purification processes of AuNPRs.

The obtained AuNPRs were subsequently pegylated to increase their stabilization in physiological media by mixing them with the same amount of PEG as AuNPRs obtained. This PEG was dissolved in a solution of NaBH_4_ (pH = 12) and incubated with gold nanoprisms in the ultrasounds for 30 minutes at 60 °C. A centrifugation step at 5000 g and 9 °C for 15 minutes as well as a gel electrophoresis process were conducted for the final purification of AuNPRs (Scheme 2).

### 2.4. Conjugation of PEGylated AuNPRs to TSLs

To carry out this conjugation it is necessary to previously activate the COOH groups of pegylated AuNPRs to which the oleylamine will be attached through their NH_2_-groups. For this activation, EDC (3·10^−3^ M) and sulfo-NHS (7·10^−3^ M) solutions were prepared in MES 10 mM pH 6. Equals parts of EDC and sulfo-NHS were mixed and incubated for 3 minutes at room temperature. Afterward, this mixture was mixed with the same volume of AuNPRs dissolved in MES 10 mM pH 6. After 30 minutes of incubation at 37 °C with agitation, three centrifugation steps of 2800 rcf at 4 °C for 9 minutes were carried out so as to purify COOH-activated AuNPRs. Finally, this solution was mixed with TSLs and left to incubate for 1 hour at 37 °C with agitation. Once again three centrifugation steps of 2800 rcf at 4 °C for 9 minutes were carried out so as to purify pegylated AuNPRs.

### 2.5. Synthesis of BioSi@NPs and co-encapsulated BioSi@NPs

For the synthesis of silica nanoparticles, we have employed a slightly modified biomimetic approach described by Jackson *et al.*^30^ in which polyethylenimine (PEI) was used as a biocompatible cationic surfactant.

In this approach, 400 μL of phosphate buffer (0.1 M, pH 8.0) and 50 μL of PEI *M*_W_ 1300 Da 10% (pH 8.0) were mixed and incubated in agitation and at room temperature for 15 minutes. During this incubation, 154 μL of the sol–gel precursor tetramethylorthosilicate, TMOS, (1 M) were hydrolyzed in 1 mL HCl (1 mM) and vortexed for 30 seconds. Subsequently, 100 μL of the hydrolyzed TMOS were added to the previous mixture. The final solution was left incubating for 5 minutes at room temperature without moving. BioSi@NPs were purified by a centrifugation process of 13.500 rpm for 5 minutes. Supernantant was discarded while the pellet was resuspended in 400 μL of phosphate buffer (0.1 M, pH 8.0). Eventually, in order to improve the monodispersity of final BioSi@NPs, they were sonicated.

For the entrapment of TSLs and AuNPRs within BioSi@NPs, the same procedure was carried out, although in this case 40 μL of TSLs of DPPC (1 mM) and 44 μL of AuNPRs were added to the initial 400 μL of phosphate buffer (0.1 M, pH 8.0).

### 2.6. Dynamic light scattering and zeta potential measurements

The Dynamic Light Scattering (DLS) technique was employed to analyze the size, polydispersion, and Zeta Potential of the samples. A Malvern Zetasizer Nano-ZS instrument from Worcestershire, UK, equipped with a monochromatic coherent 4 mM Helium–Neon laser (*λ* = 633 nm), was used for this purpose. Size measurements were conducted in triplicate using disposable cuvettes at an angle of 173° and at room temperature. Zeta potential measurements were carried out under the same conditions, employing specific folded capillary cells designed for Zeta Potential assessments.

### 2.7. Electron microscopy images

A JEM-1400 Plus transmission electron microscope (Tokyo, JP) was employed to obtain transmission electron microscopy (TEM) images. The microscope was fitted with a Gatan ORIUS camera and operated at 120 kV. Sample preparation involved placing a droplet of the samples onto 300-mesh copper grids coated with carbon acquired from Ted Pella, Inc (California, USA). Subsequently, the prepared samples were left to air-dry at room temperature.

A Inspect F50 scanning electron microscope (Eindhoven, NL) was used to capture scanning electron microscopy (SEM) images. This microscope was equipped with an INCA PensaFETx3 EDX system and operated at 200 kV. Sample preparation included depositing a drop of the samples onto a silicon wafer acquired from Ted Pella, Inc (California, USA) and, subsequently, samples were left to dry at room temperature.

### 2.8. UV-vis spectra measurements

The analysis of the plasmonic band of AuNPRs was conducted through UV-Visible-NIR spectroscopy. A Jasco V670 spectrophotometer (Madrid, ES) was employed for this purpose. The measurements were performed in triplicate using 10 mm path length quartz cuvettes at room temperature. Background intensity signals were also consistently monitored and subtracted as needed.

### 2.9. Fluorescence measurements

Fluorescence experiments were carried out with a PerkinElmer LS 55 Spectrofluorometer (Waltham, USA). The fluorescence of the hydrophilic drug model CF (*λ*_ex_ = 450 nm, *λ*_em_ = 480–600 nm) was measured in the controlled release assays and in the encapsulation efficiency experiments of BioSi@NPs. Whereas scattered light measurements were carried out in a PTI-QuantaMaster Spectrofluorometer (Birmingham, USA) fitted with a Peltier cell holder. This scattered light (area or point measure) was collected in order to estimate the transition temperature of TSLs (*λ*_ex_ = 430 nm, *λ*_em_ = 430 nm or 425–435 nm). In both cases, measurements were performed in triplicate using quartz cuvettes with a path length of 10 mm. Continuous monitoring of background intensity signals were consistently monitored and subtracted as needed.

### 2.10. Fluorescence anisotropy measurements

The thermotropic properties of TSLs were examined, in addition to scattered light experiments, by observing changes in anisotropy depending on temperature. In this sense, first of all, the fluorescent probe DPH (*λ*_ex_ = 360 nm, *λ*_em_ = 370–550 nm) was incorporated into TSLs membranes at 50 °C following the procedure outlined in Rubio-Camacho *et al.*^24^ Subsequently, once located within lipid vesicles and according to the definition of steady-state anisotropy 〈*r*〉, fluorescence intensities of DPH were measured with both excitation and emission polarizers in a vertical position (*I*_VV_) and with the excitation polarizer vertical and emission polarizer horizontal (*I*_VH_):〈r〉 = (*I*_VV_ − *GI*_VH_)/(*I*_VV_ + 2*GI*_VH_)

Measurements were performed using a Cary Eclipse Fluorescence Spectrometer (Santa Clara, USA). Excitation occurred at 360 nm while emission was recorded at 430 nm. Regarding transmissivity bias introduced by the spectrofluorometer, they were rectified using the *G* factor described as *G* = *I*_HV_/*I*_HH_.

### 2.11. NIR irradiation measurements

CF release experiments by infrared irradiation were carried out using a 1064 nm laser system (Laser Quantum, mpc6000/Ventus 1064). Samples were placed into 10 mm path length quartz cuvettes and stirred to achieve a uniform temperature distribution. Different nanoformulations (with and without AuNPRs) were irradiated for 30 seconds using a laser power intensity of 1.5 W. Nonetheless, different time of irradiation (0, 30, 60 and 120 seconds) and laser powers (0, 0.004, 0.3, 0.75, 1.5 and 2.6 W, which are equivalent to power densities of ∼0, 0.147, 4.25, 3.82, 2.98 and 3.31 W cm^−2^ respectively) were also tested. The photothermal effect (temperature increase, Δ*T*) of the different nanostructures fabricated in this study is shown in Table S1.† In all cases the *T*_m_ of the TSLs was exceeded.

## 3 Results and discussion

### 3.1. Characterization of TSLs, AuNPRs and BioSi@NPs

In order to develop these multifunctional nanoformulations, the first step was to synthesize and characterize their components separately.

#### 3.1.1 Thermosensitive liposomes

TSLs of DPPC were prepared as described in the previous section.^24^ These vesicles were characterized by DLS and TEM exhibiting well-dispersed spherical-shaped (Fig. S1a†), a hydrodynamic diameter around 167 nm and a polydispersity value of 0.17, which evidences a narrow distribution size (Table 1).

**Table 1:** Hydrodynamic diameter (*d*) and polydispersity index (PDI) of TSLs, AuNPRs and BioSi@NPs

	TSLs	AuNPRs	TSLs + AuNPRs	BioSi@NPs	BioSi@NPs + TSLs + AuNPRs
*d* (nm)	166.9 ± 2.2	90.4 ± 0.3	235.4 ± 2.5	1775.7 ± 75.8^a^	1322.8 ± 70.4^a^
PDI	0.167 ± 0.004	0.284 ± 0.008	0.204 ± 0.011	0.211 ± 0.016	0.291 ± 0.024

a Two populations were found, one at 500 nm and another one at 2 μm.

The thermal behaviour of TSLs was initially explored through the light scattered by the TSLs at different temperatures, considering that gel phase bilayers exhibit more light scattering than fluid ones.^40^ The experiment was conducted directly in the fluorometer as described in methods, employing the same wavelength for both emission and excitation monochromators and recording the scattered light at a 90° angle to the incident light. As depicted in Fig. S1b,† a notable decrease in the scattered light occurred around 41 °C, coinciding with the transition temperature reported in the literature for this lipid. This outcome was validated through fluorescence anisotropy measurements employing the fluorescent probe DPH previously integrated within lipid bilayers. The graph illustrating the steady-state fluorescence anisotropy < r> of the probe in relation to temperature displayed a well-defined sinusoidal pattern, indicating a pronounced transition in anisotropy values around 41 °C as is the case with the scattered light (Fig. S1c†).

#### 3.1.2 Gold nanoprisms

**Fig. 1:**
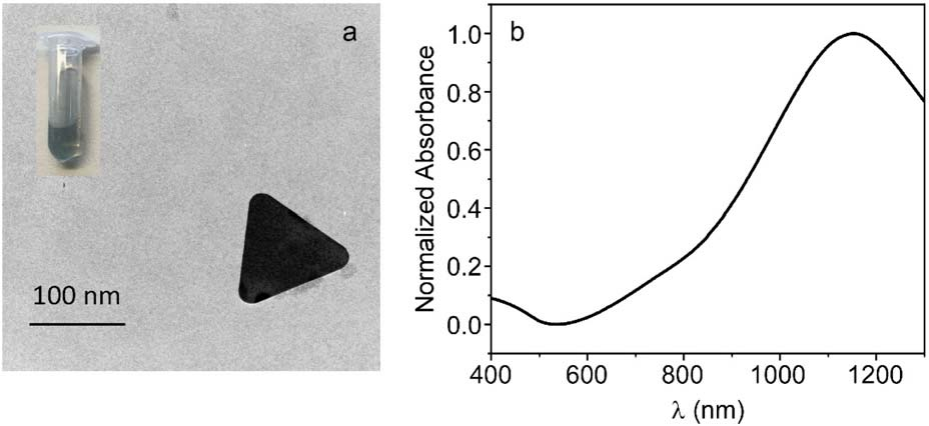
(a) Transmission electron microscopy (TEM) image showing the triangular shape of the AuNPRs synthesized in the work and digital image showing the green colour of the solution. (b) Normalized absorption spectrum of AuNPRs in phosphate buffer.

AuNPRs were synthesized and further stabilized by conjugation with heterobifunctional (HS-PEG-COOH) PEG using a surfactant-free protocol that is described in Experimental section.^37,38^ These nanoprisms were subsequently characterized obtaining hydrodynamic sizes around 90 nm with a polydispersity index of 0.28 (Table 1). Regarding AuNPRs morphology, TEM images were acquired which show triangular shapes for these gold nanoprisms (Fig. 1a).

As described in Introduction, the position of the plasmonic peak of gold nanoparticles is influenced by several factors and is possible tune and customize it by modulating the nanoparticle shape or geometry.^11,12^ The previously synthesized AuNPRs exhibit triangular shape, suggesting that their plasmonic band will be located in the NIR region, as occurs with all gold nanoparticles that grow asymmetrically forming anisotropic structures.^12^ Nonetheless, the absorbance of these nanoprisms was collected from the visible to the infrared region of the electromagnetic spectrum (400 and 1300 nm). As shown in Fig. 1b, AuNRPs exhibit a maximum peak of absorption at ∼1150 nm, which coincides with the green color of AuNPRs suspension reported in the literature for plasmonic bands located in the NIR region of the electromagnetic spectrum (see image in Fig. 1a).

#### 3.1.3 Biomimetic silica nanoparticles

BioSi@NPs were synthesized as described in Experimental section, and characterized by using DLS and SEM techniques. It should be noted that due to the size of the nanoparticles and their concentration, measurements were slightly difficult to obtain for that reason a previous dilution step (1 : 30 in milliQ water) followed by sonication (1 min) was necessary before measuring.

**Fig. 2:**
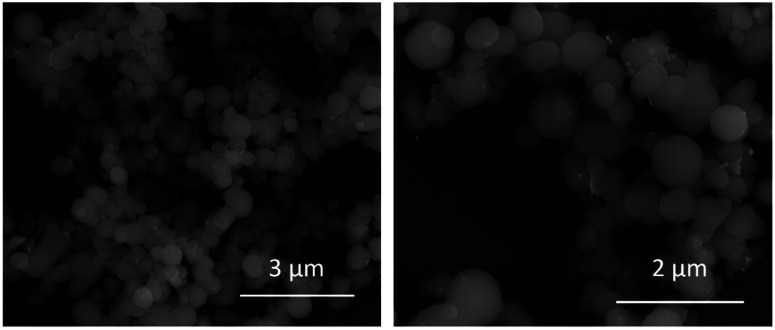
Scanning electron microscopy (SEM) images at different magnification of BioSi@NPs.

SEM images shown spherical biomimetic silica nanoparticles with a more or less homogeneous size around 500 nm, which form larger aggregates of different sizes (Fig. 2). These results were consistent with dynamic light scattering measurements which show an average hydrodynamic diameter of 1776 nm with 2 population distribution profile of 500 nm and 2 μm (Table 1). The first one coincided with the particle size obtained in scanning electron microscopy images of BioSi@NPs, while the second one would correspond to the aggregates of biomimetic silica nanoparticles which have not disaggregated during sonication.

After synthesizing and characterizing the different components, multifunctional nanoformulations were prepared. In this regard, two strategies were considered, which will be described below.

### 3.2. Covalent bonding strategy

#### 3.2.1 Characterization of PEGylated AuNPRs to TSLs

In the first approach, PEG-AuNPRs were covalently attached to the lipid membrane of TSLs, since their higher size and hydrophilic character discards their encapsulation in the liposome aqueous core, as well as their incorporation within the lipid bilayer.

**Fig. 3:**
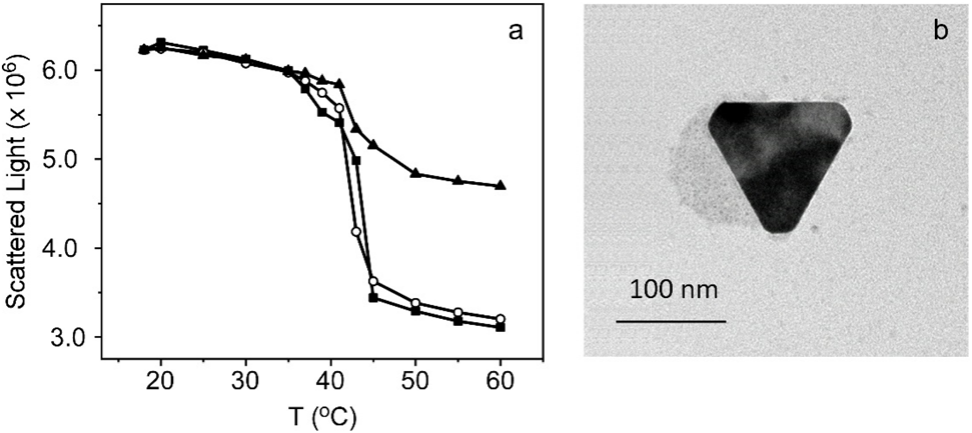
(a) Area of scattered light of TSLs (black squares), TSLs with oleylamine (circles) and TSLs with oleylamine to which AuNPRs have been attached (black triangles) as a function of temperature (20–60 °C). Note that the value recovered at 20 °C was normalized for the three samples. (b) Transmission electron microscopy (TEM) image of TSLs of DPPC 1 mM to which AuNPRs have been attached.

To bound both components, oleylamine, an unsaturated amino fatty acid with an amine group, was previously incorporated within the lipid membranes of TSLs. The incorporation of this compound, in addition to facilitating covalent bonding with AuNPRs, provides liposomal vesicles with positive charge. In fact, we confirmed its incorporation by measuring their Zeta Potential, which shows a considerable increase from 7.1 to 36.8 mV. As oleylamine is an unsaturated amino fatty acid which is incorporated into lipid bilayers during the formation of TSLs, light scattering experiments were conducted so as to explore if the thermal behavior of lipid membranes was affected by its incorporation. This experiment was carried out as previously done during the characterization of TSLs, but measuring the scattered light area (425–435 nm, at a 90° angle). The results obtained show similar behavior to unmodified TSLs, which suggests that oleylamine incorporation does not alter their thermotropic behavior (Fig. 3a).

Therefore, taking advantage of the carboxyl groups of the PEG used to stabilize the AuNPRs, as well as the amino group of the oleylamine-modified TSLs, we covalently attached gold nanoprisms to lipid vesicles. To this end, we have employed the water-soluble crosslinker EDC, capable of establishing amide bonds. This carbodiimide provides the most popular and commonly used method for crosslinking to carboxylic acids.^41^ EDC reacts with carboxylic acid groups to form an unstable intermediate which is rapidly attacked by primary amino groups, forming an amide bond with the original carboxyl group.^41^ It should be noted that sulfo-NHS is often included in EDC coupling protocols in order to improve its efficiency.

The binding of AuNPRs to TSLs resulted in nanohybrids that were subsequently characterized trough DLS (Table 1). The results revealed a growth in the hydrodynamic diameter of liposomes, increasing from 167 nm to 235 nm, a difference approximately equivalent to the size of AuNPRs. This outcome suggests that nanoprisms have been successfully attached to TSLs. Nevertheless, in order to verify this hypothesis, TEM images were acquired in which it can be seen spherical shapes, corresponding to liposomes, on which AuNPRs are deposited, confirming the results previously obtained (Fig. 3b). Regarding the thermal behavior of the nanohybrids, it was also analyzed through light scattering area measurements (425–435 nm, at a 90° angle). The Fig. 3a evidences a drop of the scattered light around 41 °C, suggesting that lipids are correctly packed and undergo phase transition cooperatively after covalent binding with AuNPRs. The major difference compared to unmodified TSLs and oleylamine-modified TSLs lies in the increase in scattered light observed at temperatures above the phospholipids melting point (*T*_m_) for TSLs covalently bound to AuNPRs. This increase can be attributed to the heightened light scattering induced by gold nanoprisms. Consequently, the conjugation of AuNPRs to TSLs does not appear to modify the thermal behavior, thereby enabling the controlled release of drugs from liposomes.

#### 3.2.2 Encapsulation of fluorescent probe and release assays

Hereinafter, the capacity to encapsulate drugs and release their content through photothermal therapy was explored. To this end, we have employed the fluorescent probe carboxyfluorescein encapsulated within the aqueous cavity of TSLs following the procedure outlined in Experimental section. This hydrophilic compound was selected for the release assays due to its compelling photophysical properties according to which at elevated concentrations most of its fluorescence is quenched, primarily as a result of both a dimerization process leading to non-fluorescent compounds and Förster Resonance Energy Transfer (FRET) between formed dimers and monomers.^42^ Given the entirely reversible nature of this process, it is possible to assess the release of CF from TSLs due to the increase in the fluorescence signal when it escapes from the liposomes and is diluted in the external medium.

**Fig. 4:**
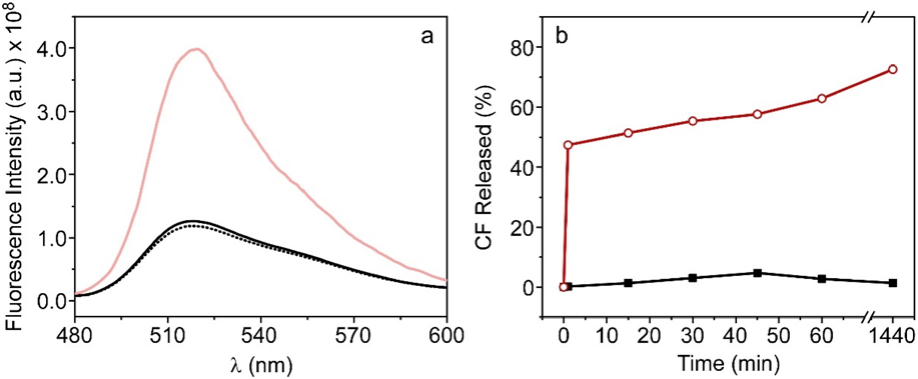
(a) Fluorescence emission spectra of CF encapsulated in TSLs with AuNPRs attached to their surface before (black dot) and after (red line) irradiation, and without covalently bonded AuNPRs after irradiation (black line). (b) Release kinetics of CF in % as a function of time (0–1440 min) from TSLs in both absence (black squares) and presence (red circles) of covalently bonded AuNPRs.

The release of the encapsulated CF is induced by altering the fluidity of the TSL bilayer which takes place during the gel-fluid phase transition. To achieve this, we have employed the heat generated by the AuNPRs covalently bonded to them upon exposure to a laser. It should be noted that these gold nanoprisms stand out for exhibiting a plasmonic band in the infrared region of the electromagnetic spectrum, around 1150 nm (Fig. 1b). Hence, a continuous wave 1064 nm laser system was applied for the release assays. The experiments were conducted following the procedures described in Experimental section, involving irradiation for 30 seconds at a power of 1.5 W. The fluorescence spectrum of CF (480–600 nm) was recorded both before and after the irradiation of the samples so as to compare their fluorescence. In Fig. 4a, a rise in the fluorescence of the probe is depicted after irradiation, providing evidence of its release from lipid vesicles. As a negative control, a similar experiment was carried out with TSLs lacking covalently bound gold nanoprisms, revealing no discernible difference in their fluorescence signal after irradiation, whichs confirm that AuNPRs were ultimately responsible for the release (Fig. 4a).

Fluorescence was also collected at different times to analyse the release profile of the nanoformulation. For this purpose, seven identical samples were irradiated as previously described (30 seconds at 1.5 W) and the fluorescence was collected at 0, 1, 15, 30, 45, 60 and 1440 min. The percentage of CF release was calculated based on changes in the fluorescence signal at 520 nm after the addition of detergent Tween20. Results shown in Fig. 4b indicate that, in the absence of AuNPRs, there is not leakage of CF after laser irradiation, evidencing that the probe remains confined within the TSLs. Nonetheless, in the nanohybrids, there is a rapid release of CF, particularly in the initial minutes, during which approximately the 50% of the compound is released. This result highlights the capacity of the hybrid nanoparticle to transport and release compounds triggered by photothermia within the first minutes, maintaining thereafter a sustained release over time.

**Fig. 5:**
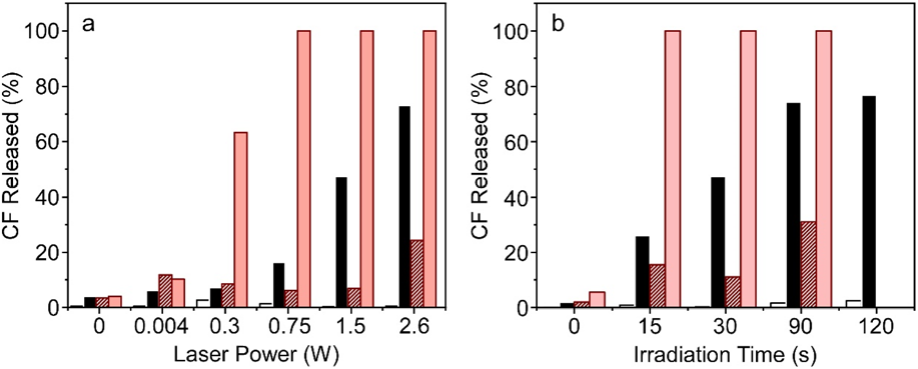
Effect of (a) laser power (time of irradiation 30 seconds) and (b) time of irradiation (laser power of 1.5 W) on the release of CF within the first minutes of TSLs (white), TSLs + AuNPRs (black), biomimetic silica NPs + TSLs (striped red) and biomimetic silica NPs + TSLs + AuNPRs (red).

In the Introduction, the importance of both an immediate and sustained release over time has been discussed. In this regard, the ability of this nanoformulation to modulate its release kinetics based on parameters such as irradiation power and exposure time has been investigated. Taking into account that the aforementioned experiments were performed at a laser power of 1.5 W (80%) for 30 seconds, we proceed to investigate and compare the percentage of CF released at both lower and higher laser power settings, as well as different exposure times. Fluorescence signal was collected at 520 nm after laser irradiation. Liposomes, wherein CF was encapsulated without being covalently bound to gold nanoprisms, were also prepared as negative controls. Immediate release percentages lower than those previously obtained were reached by employing lower laser powers and exposure times. The opposite effect was also achieved, higher CF release rates were observed with increased laser powers and longer exposure times (Fig. 5a and b). This demonstrates a clear relationship between release percentage and the applied power or exposure time, providing the ability to precisely regulate the release kinetics of the fluorescent probe according to the desired outcome: immediate or sustained release profile. It is noteworthy that, once again, this effect is specifically observed when gold nanoprisms are covalently bound to the thermosensitive liposomes.

### 3.3. Coencapsulation strategy

#### 3.3.1 Characterization of BioSi@NPs containing TSLs and AuNPRs

The second nanoformulation explored in this study involved the coencapsulation of thermosensitive liposomes and gold nanoprisms within silica nanoparticles. These nanoparticles were selected for their structure, which allows efficient drug loading and controlled drug release. Since conventional synthesis strategies have numerous drawbacks when it comes to encapsulating biological compounds, in this study we have employed a biomimetic silica synthesis technique that operates under gently physical and environmentally friendly conditions as is described in Experimental section. Furthermore, this strategy, apart from being biocompatible, avoids covalent binding and modification of both thermosensitive liposomes and gold nanoprisms.

**Fig. 6:**
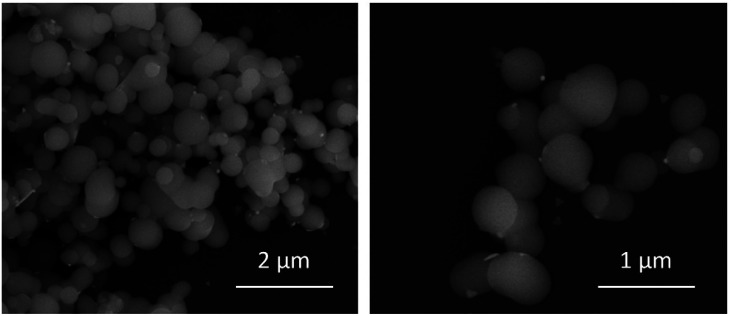
Scanning electron microscopy (SEM) images at different magnification of BioSi@NPs containing encapsulated TSLs and AuNPRs.

After synthesizing biomimetic silica nanoparticles containing TSLs and AuNPRs, we proceed to characterize them through dynamic light scattering (Table 1) and scanning electron microscopy (Fig. 6). It is crucial to note that for the acquisition of biomimetic silica nanoparticles characterization measurements, a preliminary step which involve their dilution and subsequent sonication was necessary. The resulting nanoparticles exhibit a spherical morphology with sizes around 500 nm, which confirms that the entrapment of thermosensitive liposomes and gold nanoprisms does not disrupt the formation of biomimetic silica nanoparticles. The images also reveal the presence of some unencapsulated AuNPRs in close proximity to the BioSi@NPs. However, considering the initial concentration of added AuNPRs, it can be inferred that the majority of the AuNPRs are located within the biomimetic silica nanoparticles.

#### 3.3.2 Biomimetic silica nanoparticles as drug carriers

**Fig. 7:**
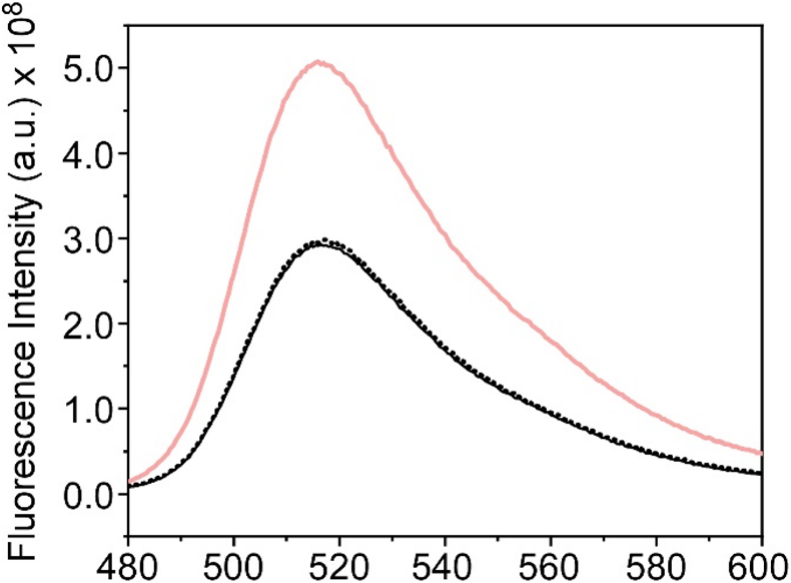
Fluorescence emission spectra of CF encapsulated within TSLs (red) and TSLs entrapped inside BioSi@NPs, both in the presence (black) and absence (black dot) of gold nanoprisms, after Tween20 addition.

As stated in the Introduction, biomimetic silica nanoparticles exhibit a high encapsulation efficiency. To assess this encapsulation efficiency, we have employed the fluorescence emitted by the encapsulated CF within the TSLs upon the addition of Tween20. The fluorescence signal obtained for the fluorescent probe encapsulated within the thermosensitive liposomes after the addition of the detergent was compared with the signal obtained for CF of TSLs encapsulated within the BioSi@NPs, both in the presence and absence of AuNPRs. As is shown in Fig. 7, the fluorescence spectrum of CF was observed in all the cases. The only distinction among them lied in the higher fluorescence signal obtained when TSLs were not encapsulated inside silica nanoparticles. This difference allowed us to determine an encapsulation efficiency of approximately 44% for liposomal vesicles within the BioSi@NPs. It was noteworthy that the fluorescence emission spectrum of CF remained unaltered in the presence of AuNPRs in the BioSi@NPs, indicating the absence of non-specific interactions with AuNPRs.

**Fig. 8:**
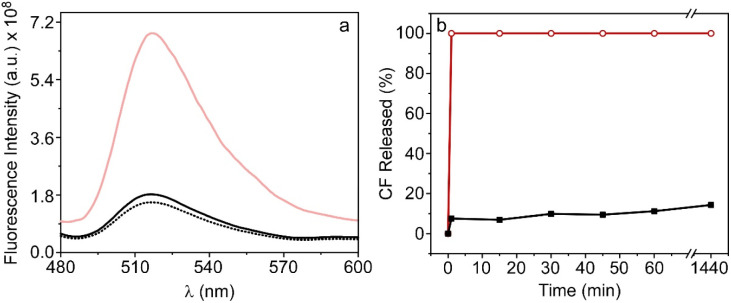
(a) Fluorescence emission spectra of CF encapsulated in liposomes coencapsulated with AuNPRs within BioSi@NPs before (black dot) and after (red line) irradiation, and without AuNPRs after irradiation (black line). (b) Release kinetics of CF in % as a function of time (0–1440 min) from TSLs in both absence (black squares) and presence (red circles) of AuNPRs coencapsulated within biomimetic silica nanoprisms.

After confirming the encapsulation of TSLs and AuNPRs within the silica nanoparticles, we proceeded to evaluate the capability of this novel nanostructure to encapsulate and release drugs through photothermal therapy as previously conducted for the TSLs to which gold nanoprisms were covalently attached. For this purpose, the fluorescent probe carboxyfluorescein was once again employed, encapsulating it within TSLs coencapsulated with AuNPRs in BioSi@NPs. As in the previous case, the fluorescence emission spectrum of CF was recorded before and after irradiating this new nanostructure using the infrared laser (30 seconds at a power of 1.5 W). The results obtained exhibit similar behavior to TSLs covalently linked to AuNPRs: a pronounced increase in the fluorescence signal of the probe after irradiating the nanostructure, exclusively in the presence of AuNPRs (Fig. 8a).

The release kinetics were also studied by measuring the fluorescence signal of CF at 520 nm at different time points after the addition of the detergent Tween20 as previously done. The results indicated the release of nearly 100% of the probe after the exposure to laser irradiation (Fig. 8b). It should be noted that this effect was observed exclusively in the presence of AuNPRs, suggesting once again that the liberation is likely attributed to the heat generated by AuNPRs.

These results highlight the capability of this new system not only to encapsulate and release drugs through photothermal therapy but also to achieve a significantly faster release compared to TSLs covalently bound to AuNPRs. This effect could be attributed to the biomimetic silica structure superior heat retention, preserving a larger amount of heat generated by the encapsulated AuNPRs after irradiation. This promotes increased fluidity in the liposomal membranes, thereby facilitating a more pronounced release of the CF contained within them.

In an attempt to study if we were able to modulate the release kinetics of this nanostructure, both the laser power and the irradiation time were modified separately. No decrease in release was observed when reducing the irradiation time while maintaining the laser power at 1.5 W (Fig. 5b). Lower release percentages were only noticeable when the laser power was decreased maintaining the irradiation time at 30 seconds (Fig. 5a). It could be reasonably assumed that decreasing both the laser power (<1.5 W) and the irradiation time (<30 s) would result in a reduction of the release kinetics.

## 4 Conclusions

The study aimed to develop multifunctional nanoformulations involving TSLs, AuNPRs and BioSi@NPs. After synthesizing and characterizing individual components, two strategies were employed to create the nanoformulations. In the first approach, AuNPRs were covalently bonded to TSLs through a crosslinking method, resulting in successful attachment without altering the thermal behavior of TSLs. This hybrid nanostructure demonstrated the controlled release of the encapsulated fluorescent compound carboxyfluorescein through photothermal therapy by irradiation with a 1064 nm laser.

The second strategy involved coencapsulation of TSLs and AuNPRs within BioSi@NPs. This novel nanostructure exhibited efficient encapsulation of TSLs and AuNPRs of approximately 44%. As for the previous nanoformulation, the release of CF trapped in the nanohybrids was triggered by infrared irradiation. The release kinetics of BioSi@NPs were faster compared to TSLs covalently bound to AuNPRs, potentially attributed to the superior heat retention of the biomimetic silica structure.

Moreover, this study demonstrated the ability to modulate the release kinetics of these nanoformulations by adjusting laser power and irradiation time. Lower laser power and shorter exposure times resulted in decreased release percentages, highlighting the precise control over the release profile.

In summary, the multifunctional nanoformulations developed in this study show promising potential for applications in controlled drug delivery through photothermal therapy, offering versatility and tunability in release kinetics based on specific therapeutic requirements.

## Data availability

All data generated or analyzed during this study are included in this published article and its ESI files. The data supporting this article have been included as part of the ESI.†

## Author contributions

Conceptualization: C. C.-A., J. M. F., C. R. M.; data curation: M. R.-C.; formal analysis: M. R.-C.; funding acquisition: M. J. M. T., J. M. F., C. R. M.; investigation: M. R.-C.; methodology: M. R.-C., C. C.-A., B. T.-H.; project administration: M. J. M. T., J. M. F., C. R. M.; resources: M. J. M. T., J. M. F., C. R. M.; supervision: C. C.-A., M. J. M. T., J. M. F., C. R. M.; validation: M. R.-C.; visualization: M. R.-C., M. J. M. T.; writing – original draft: M. R.-C.; writing – review & editing: M. R.-C., C. C.-A., B. T.-H., M. J. M. T., J. M. F., C. R. M.

## Conflicts of interest

There are no conflicts to declare.

## Supplementary Material

RA-014-D4RA03359K-s001
