# Supplementary material for: Harnessing the power of thermosensitive liposomes with gold nanoprisms and silica for controlled drug delivery in combined chemotherapy and phototherapy
Source: RSC Adv. 2024 Jul 22;14(32):23073–82. doi: 10.1039/d4ra03359k (PMC11261576; doi:10.1039/d4ra03359k)
Supplement: RA-014-D4RA03359K-s001 [file RA-014-D4RA03359K-s001.pdf]

Electronic Supplementary Material (ESI) for RSC Advances.
This journal is © The Royal Society of Chemistry 2024

Supporting Information

Harnessing the Power of Thermosensitive Liposomes with Gold Nanoprisms and Silica for Controlled Drug Delivery in Combined Chemotherapy and Phototherapy

Marta Rubio-Camacho,[*a] Carlos Cuestas-Ayllón,[b] Beatriz Torres-Herrero,[b] María José Martínez-Tomé,[a] Jesus M. de la Fuente[b] and C. Reyes Mateo[a]

[a]Instituto de Investigación, Desarrollo e Innovación en Biotecnología Sanitaria de Elche (IDiBE), Universidad Miguel Hernández (UMH), c/ Avenida de la Universidad de Elche, s/n, 03202 Elche, Alicante, Spain

[b]Instituto de Nanociencia y Ciencia de Materiales de Aragón, CSIC-Universidad de Zaragoza, c/ Pedro Cerbuna s/n, 50009 Zaragoza, Spain & CIBER-BBN

Correspondence: Marta Rubio-Camacho

Instituto de Investigación, Desarrollo e Innovación en Biotecnología Sanitaria de Elche (IDiBE), Universidad Miguel Hernández (UMH), c/ Avenida de la Universidad de Elche, s/n, 03202 Elche, Alicante, Spain

Tel: +34 965 222479

Email: marta.rubioc@umh.es

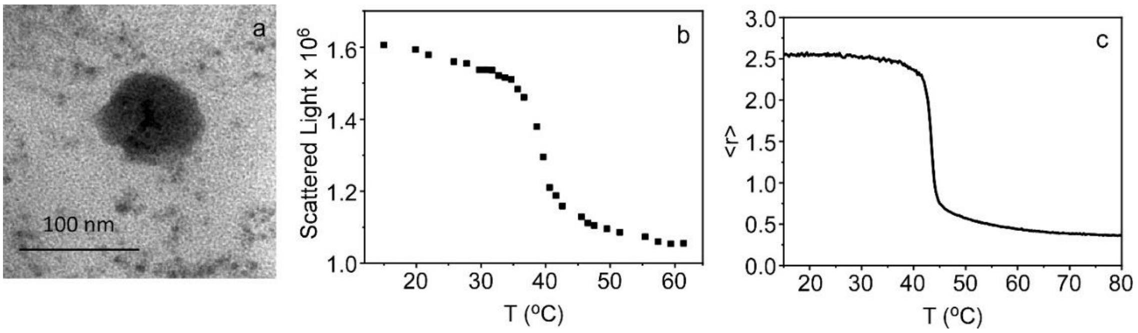

Fig. S1. (a) Transmission electron microscopy (TEM) image of TSLs composed of DPPC, effect of temperature on (b) the scattered light and on (c) the fluorescence anisotropy of TSLs.

Table S1. Photothermal effect (temperature increase, ΔT) of the different nanostructures fabricated irradiating at a laser power of 1.5 W for 60 seconds.

**SAMPLE**	H_2O	TSLs	NPRs	AuNPRs covalently bound to TSLs	†AuNPRs and TSLs coencapsulated in BioSi@NPs
**ΔT (°C)**	3.9 ± 0.9	9.0 ± 0.7	59.3 ± 1.9	52.5 ± 1.3	20.1 ± 3.4

† The actual temperature reached inside the structure of the BioSi@NPs after irradiation could not be determined, we only determined the temperature in the solution.
